# Development of an ELISA Assay for the Determination of SARS-CoV-2 Protein Subunit Vaccine Antigen Content

**DOI:** 10.3390/v15010062

**Published:** 2022-12-24

**Authors:** Lu Han, Chaoqiang An, Dong Liu, Zejun Wang, Lianlian Bian, Qian He, Jianyang Liu, Qian Wang, Mingchen Liu, Qunying Mao, Taijun Hang, Aiping Wang, Fan Gao, Dejiang Tan, Zhenglun Liang

**Affiliations:** 1 NHC Key Laboratory of Research on Quality and Standardization of Biotech Products, NMPA Key Laboratory for Quality Research and Evaluation of Biological Products, National Institutes for Food and Drug Control, Beijing 102600, China; 2 College of Pharmacy, China Pharmaceutical University, Nanjing 210000, China; 3 Beijing Minhai Biotechnology Co., Ltd., Beijing 102629, China; 4 Changchun Institute of Biological Products Co., Ltd., Changchun 130062, China; 5 Wuhan Institute of Biological Products Co., Ltd., Wuhan 430070, China; 6 College of Life Sciences, Zheng Zhou University, Zhengzhou 450001, China

**Keywords:** severe acute respiratory syndrome coronavirus 2, SARS-CoV-2, coronavirus disease, protein subunit vaccine, antigen content, analytical quality by design (AQbD)

## Abstract

The severe acute respiratory syndrome coronavirus 2 (SARS-CoV-2) protein subunit vaccine is one of the mainstream technology platforms for the development of COVID-19 vaccines, and most R&D units use the receptor-binding domain (RBD) or spike (S) protein as the main target antigen. The complexity of vaccine design, sequence, and expression systems makes it urgent to establish common antigen assays to facilitate vaccine development. In this study, we report the development of a double-antibody sandwich enzyme-linked immunosorbent assay (ELISA) to determine the antigen content of SARS-CoV-2 protein subunit vaccines based on the United States Pharmacopeia <1220> and ICH (international conference on harmonization) Q14 and Q2 (R2) requirements. A monoclonal antibody (mAb), 20D8, was identified as the detection antibody based on its high RBD binding activity (EC50 = 8.4 ng/mL), broad-spectrum anti-variant neutralizing activity (EC50: 2.7–9.8 ng/mL for pseudovirus and EC50: 9.6–127 ng/mL for authentic virus), good in vivo protection, and a recognized linear RBD epitope (369–379 aa). A porcine anti-RBD polyclonal antibody was selected as the coating antibody. Assay performance met the requirements of the analytical target profile with an accuracy and precision of ≥90% and adequate specificity. Within the specification range of 70–143%, the method capability index was >0.96; the misjudgment probability was <0.39%. The method successfully detected SARS-CoV-2 protein subunit vaccine antigens (RBD or S protein sequences in Alpha, Beta, Gamma, or Delta variants) obtained from five different manufacturers. Thus, we present a new robust, reliable, and general method for measuring the antigenic content of SARS-CoV-2 protein subunit vaccines. In addition to currently marketed and emergency vaccines, it is suitable for vaccines in development containing antigens derived from pre-Omicron mutant strains.

## 1. Introduction

Coronavirus disease 2019 (COVID-19) is caused by severe acute respiratory syndrome coronavirus 2 (SARS-CoV-2) infection, which rapidly spread around the world and continues to pose a considerable risk to public health [1,2]. The development of safe, effective, and quality-controlled vaccines is one of the main tools for reducing the risk of SARS-CoV-2 transmission. Protein subunit vaccines can be designed to be multi-component by including subunits from different variants of concern (VOCs) and mass-produced without the need for viruses, making them the leading technology platform for SARS-CoV-2 vaccine development. Protein subunit vaccines ZF2001 produced by Anhui Zhifei Longcom Biopharmaceutical Co., Ltd. (Longcom, Anhui, China) and Nvx-CoV2373 produced by Novavax (Gaithersburg, MD, USA) have already been approved for SARS-CoV-2 [3,4,5]. As of 1 November 2022, a total of 172 SARS-CoV-2 vaccines based on different technologies are in clinical trials, of which 55 (32%) are protein subunit vaccines [6]. SARS-CoV-2 enters cells through an interaction between the receptor-binding domain (RBD) of the viral spike (S) protein and the host cell. Thus, SARS-CoV-2 protein vaccines have been designed and developed using the RBD or S protein as an antigen. It is crucial to accurately quantify the RBD in a vaccine to evaluate its immunogenicity, safety, and efficacy. However, differences in vaccine sequence, design, and expression system pose significant challenges for regulatory authorities worldwide [7,8,9]. Our aim was to establish a standardized universal RBD quantification method for SARS-CoV-2 protein subunit vaccines, which is essential to accelerate vaccine development and application.

In 2004, the U.S. Food and Drug Administration (FDA) introduced the concept of quality by design (QbD) [10]. Subsequently, the concept was applied to the development of analytical methods, resulting in the principle of analytical quality by design (AQbD) [11]. With this in mind, the United States Pharmacopeia (USP) released the following guidelines: <1032> on the Design and Development of Biological Assays (2013), <1033> on Biological Assay Validation (2013), and <1210> on Statistical Tools for Procedure Validation (2018) [12,13,14]. Chapter <1220> of the Analytical Procedure Life Cycle was promoted in the USP in May 2022 [15]. The International Council for Harmonization of Technical Requirements for Pharmaceuticals for Human Use (ICH) also published new guidelines on method development (Q14) and validation Q2 (R2) in March 2022 [16,17]. The concept of AQbD was further defined by proposing new terms, such as analytical target profile (ATP), risk assessment, critical method attributes (CMAs), analytical procedure parameters, and method operable design region (MODR) [18]. AQbD also emphasizes the importance of using an enhanced approach, that is, a multifactorial design of experiment (DoE), for method optimization and robustness testing [19]. Advances in the methodology described above also help guarantee assay quality. However, there are no recent reports on establishing biological assays based on the latest requirements of USP <1220>, ICH Q14, or Q2 (R2).

In this study, an ELISA method was developed based on the above-mentioned concepts of USP <1220>, ICH Q14, and Q2 (R2), and it was used to determine the antigenic content of SARS-CoV-2 protein subunit vaccines. A porcine anti-RBD polyclonal antibody developed by Zhengzhou University was used as the coating antibody. After characterizing the properties of monoclonal antibody (mAb) 20D8, it was conjugated with horseradish peroxidase (HRP) and used as the detecting antibody. The method provides a robust, reliable, and universal assay for quality control of SARS-CoV-2 protein subunit vaccines. Determination of the antigenic content of vaccines can help to solve key technical bottlenecks in the development and application of protein subunit-based SARS-CoV-2 vaccines. This method is only applicable to protein subunit vaccines. It should be noted that mRNA vaccines, adenovirus-based vaccines, and whole virus-based vaccines cannot be quantified using this method.

## 2. Materials and Methods

### 2.1. Materials

Golden hamsters (6 weeks old, male) were provided by Kunming National High-level Biosafety Primate Research Center, China, and challenged with authentic SARS-CoV-2 using the hamster model in an ABSL-4 facility. BALB/c mice (6 weeks old, female) were provided by the Wuhan Institute of Biological Products Co., LTD (Wuhan Institute, Wuhan, China) for producing mAbs. All animal studies were conducted according to relevant ethical regulations and were approved by the Institutional Animal Care and Use Committee of the Institute of Medical Biology, Chinese Academy of Medicine Sciences and Peking Union Medical College, China (approval number: DWSP202101001) and Wuhan Institute (approval number: WIBP-A II 382020001).

Carbonate buffer (pH 9.6) was used for coating, and phosphate-buffered saline (PBS) containing 20% fetal bovine serum (FBS; Cellmax, Australia) was used as a blocking solution. Samples and enzymes were diluted in PBS containing 10% FBS. Tetramethylbenzidine (TMB) and termination solution was purchased from Beijing Wantai Biological Pharmaceutical Co. (Beijing, China). A Spectra Max iD3 microplate reader was purchased from Molecular Devices (San Jose, CA, USA), and an xMark^TM^ Microplate Absorbance Spectrophotometer was purchased from Bio-Rad (Hercules, CA, USA).

### 2.2. ATP

The intended purpose of the developed method was to establish the antigen content of SARS-CoV-2 protein subunit vaccines, and thus to perform release testing of such products. The ATP was based on previous ELISA experimental results [20]. AQbD was used throughout ELISA development to ensure that the vaccines met all necessary quality requirements [21,22].

### 2.3. mAb Screening

#### 2.3.1. mAb Preparation

BALB/c mice were immunized with inactivated SARS-CoV-2 antigen and Freund’s adjuvant (Sigma-Aldrich, Saint Louis, USA). Hybridoma cells were prepared according to previous studies [23]. mAbs (14C8, 15E9, 17A7, 20D8) were screened using the RBD protein. The positive hybridoma cells screened were cloned three times and then scaled up for culture to prepare ascites. mAb-HRP was produced using a Lightning-Link^®^ antibody labeling kit (Abcam, ab102890, UK).

#### 2.3.2. RBD Binding Activity and Neutralization Assay

The binding activity of mAbs to the RBD was measured using ELISA. The 96-well enzyme immunoassay/radioimmunoassay plate was coated with 10 ng RBD protein (Longcom, Anhui, China) per well for 12 h at 4 °C. ELISA was performed as previously described [24].

Neutralization assays were performed using SARS-CoV-2 pseudoviruses and authentic viruses to determine the half-maximal inhibitory concentration (IC_50_) of mAbs against different variant strains [24].

#### 2.3.3. Challenge Trials in Hamsters

Fifteen healthy hamsters were challenged with 1 × 10^6^ tissue culture-infective dose of wild-type (WT) or B.1.351 (Beta) SARS-CoV-2 via intranasal administration. After 1 d, the hamsters were intraperitoneally injected with 0.5 mg of mAb 20D8 (*n* = 6), K8G2 (Enterovirus 71 mAb as the negative group) (*n* = 5), or PBS (as the control group) (*n* = 4). On the 5th day after the challenge, the left lung of each hamster was obtained for pathological examination, and the right lung was obtained to determine the viral load.

Lung pathology was evaluated using hematoxylin and eosin staining, as previously described [25]. Formalin-fixed paraffin-embedded sections (5 μm) were prepared and stained. The slides were evaluated by a pathologist in a double-blinded manner. Pathological scores were calculated based on a pathological scoring system established by the Institute of Medical Biology, Chinese Academy of Medical Sciences. The scoring template for SARS-CoV was based on the method described by Liu et al. [26].

On day 5 post-challenge, lung specimens were collected for viral RNA detection. Tissues were weighed, homogenized, and clarified by centrifugation at 6200× *g* for 10 min at 4 °C, and supernatants were obtained. Viral RNA was extracted using a magnetic viral nucleic acid kit (TIANGEN, Beijing, China), according to the manufacturer’s protocol. Viral genomic RNA in each sample was quantified by reverse transcription–polymerase chain reaction (RT-PCR) targeting the N gene of SARS-CoV-2. The lower limit of detection was transformed to 2500 copies/g of the lung tissue.

#### 2.3.4. Western Blot Analysis

RBD protein was mixed with 6× loading buffer, boiled at 100 °C for 15 min, separated by electrophoresis in 8% SDS polyacrylamide gels (ExpressPlus™ PAGE Gel, 10 × 8, 8%, 10 wells, Genescript, Nanjing, China), and transferred onto nitrocellulose membranes. The membranes were blocked with 5% skim milk for 2 h at room temperature and then incubated with the primary antibody (20D8 or rabbit polyclonal antibody) for 12 h at 4 °C. The membranes were then washed three times with TBST (Tris-buffered saline, 0.1% Tween 20) and incubated with an HRP-conjugated secondary antibody (ZSQB-BIO, ZB2305, ZB-5301, China) for 1 h at room temperature. The membranes were then washed three times with TBST. Finally, the immunoblot results were visualized using electrochemiluminescence (ECL) hypersensitive luminous solution (Thermo Fisher, 32209, Waltham, USA) and detected using a charge-coupled device imaging system.

#### 2.3.5. Epitope Mapping

Plates were individually coated with 0.01 μg/mL of each synthetic peptide in coating buffer (0.1 M NaHCO_3_, pH 8.6), incubated overnight at 4 °C, and then blocked with casein at 37 °C for 1 h. The plates were then incubated with 20D8 monoclonal antibody (50 μL/well diluted to 2 μg/mL) at 37 °C for 30 min. The plates were then washed five times and dried. Binding of 20D8 to the immobilized peptides was detected using an anti-mouse IgG-HRP conjugate, followed by color development using the TMB substrate. The binding efficiency was estimated by measuring the absorbance at 450/630 nm using a spectrophotometer (Bio-Rad). Epitope mapping and alanine scanning methods were performed according to the steps of the above experiment. A panel of synthetic peptides covering the entire sequences of RBD of SARS-CoV-2 were synthesized by Sangon Biotech Co., Ltd. (Shanghai, China).

### 2.4. Polyclonal Antibody Preparation

Porcine polyclonal antibodies were prepared by Zhengzhou University using the SARS-CoV-2 protein subunit vaccine (Chinese hamster ovary (CHO) cell). Porcine polyclonal antibody was purified by affinity chromatography using Protein G Sepharose 4 Fast Flow (Cytiva, 17061806, Wilmington, DE, USA).

### 2.5. National Standard

The National Standard (NS) for the first Chinese national standard for protein subunit SARS-CoV-2 vaccines (lot 300050-202101) was used at a concentration of 877,000 U/mL. It was stored at the National Institutes for Food and Drug Control (NIFDC) [27].

### 2.6. Establishment and Optimization

Owing to mAb specificity, the inherent justification of ELISA is characterized by specificity. The recombinant S proteins of SARS-CoV and the Middle East respiratory syndrome-related coronavirus (MERS-CoV) were diluted to 1 µg/mL. The specificity of the ELISA was tested using minimum essential medium (MEM), Dulbecco’s modified eagle medium (DMEM), FBS and PBS as negative controls, and the NS (0.4 U/mL) as a positive control.

DoE is an essential component of the AQbD concept [28,29]. Quality risk assessment was applied at an early stage of method development to identify the main factors affecting the method. The main factors affecting ELISA results were tested as follows: concentration (coating antibody and enzyme-labeled mAb), temperature (antigen and antibody incubation and color development), and incubation time (antigen, antibody, and color development). The concentrations of coating antibody (0.125–4 μg/mL) and enzyme-labeled mAb (0.03125–4 μg/mL) were optimized using an analytical design. Polyclonal porcine anti-SARS-CoV-2 antibodies were diluted in a coating solution to a final concentration of 100 µL/mL in a 96-well plate and incubated overnight at 2–8 °C. The plate was washed five times, dried, and 200 µL/well of blocking solution was added, followed by incubation at 37 °C for 2 h. The plate was then washed five times, dried, and 100 µL/well of NS (1 U/mL) was added, and incubation was carried out at 37 °C for 1 h. The plate was washed five times, dried, and 100 µL/well of enzyme-labeled monoclonal antibody diluent was added, followed by incubation at 37 °C for 1 h. The color was developed at 37 °C for 10 min in the dark. Termination solution (50 µL/well) was added, and the absorbance was measured at 450/630 nm.

Based on custom designs using JMP 13^®^ software (SAS Institute, Cary, NC, USA), CMAs and analytical procedure parameters were added (Table S1). According to the JMP 13^®^ software predictive profiler function, the MODR of the experiment was determined and the robustness of the method was examined. To determine the experiment’s applicable model, the NS was diluted to 1 U/mL, followed by seven 2-fold dilutions, and four independent experiments were performed to measure the antigen content.

### 2.7. ELISA Validation

#### 2.7.1. Range

To establish a range for a biological assay based on USP <1033> biological assay validation, a dilution series of standards may be used to assess the correlation between known and measured relative potency [13]. In this study, the NS was diluted to 0.5 U/mL with sample diluent, followed by six 2-fold dilutions, and 16 independent experiments were performed to measure the antigen content.

#### 2.7.2. Accuracy and Precision

The key factors affecting the accuracy and precision of the experimental method are the analyst, enzyme marker, and time. A nested design was used, with two analysts using two different enzyme markers for readings, carrying out two runs per day, with two tubes in parallel within each run, for a total of 4 days of experimentation (Figure S1). Sixteen independent experiments were performed to detect antigen content in order to have an adequate sample size.

### 2.8. ELISA Suitability Evaluation

Five VOCs of bulks of the SARS-CoV-2 protein subunit vaccines based on Alpha, Beta, Gamma, and Delta variants were supplied by five Chinese manufacturers. Four bulks were expressed in CHO cells, and one in Sf9 cells. Two of the bulks were S proteins and three were RBD proteins. A validated ELISA, developed to test the antigen content in SARS-CoV-2 protein subunit vaccines, was used to study the suitability of the five bulks of SARS-CoV-2 protein subunit vaccines. Notably, mRNA vaccines, adenovirus-based vaccines, and whole virus-based vaccines cannot be quantified using this method.

### 2.9. Statistical Analysis

JMP 13^®^ software was used for experimental design and analysis of the results using analysis of variance (ANOVA). BMV^®^ software was used to calculate the relevant method validation indicators [30]. The results of the quantitative antigen assay were statistically analyzed by applying the double parallel line method using Biostat ^®^ software (1.0).

## 3. Results

### 3.1. mAb 20D8 Exhibits Good Neutralizing Capacity against WT and Variant Strains of SARS-CoV-2

**Figure 1:**
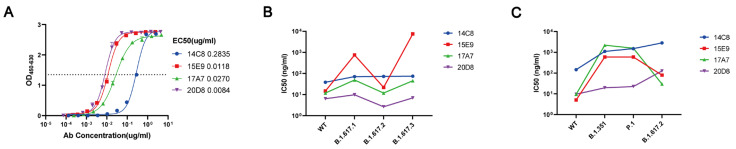
Receptor-binding domain (RBD) binding activity and neutralizing activity of four monoclonal antibodies (mAbs) against variant SARS-CoV-2 strains. (**A**) Binding activity of four mAbs to the RBD. (**B**) Neutralizing activity of four mAbs toward SARS-CoV-2 wild-type (WT) and pseudovirus variant strains (B.1.617.1, B.1.617.2, and B.1.617.3). (**C**) Neutralizing activity of four mAbs toward SARS-CoV-2 WT and variant strains (B.1.351, P.1, and B.1.617.2).

Four mAbs with high neutralizing activity against SARS-CoV-2 WT, coded 14C8, 15E9, 17A7, and 20D8, were screened from among 13 mAbs to determine their RBD binding activity and neutralizing activity against pseudoviruses and viruses. mAb 20D8 exhibited the highest RBD binding activity among the four mAbs, with a 50% maximal effect at a concentration of 8.4 ng/mL (Figure 1A). mAb 20D8 also exhibited high neutralizing activity against pseudovirus variants (B.1.617.1, B.1.617.2, and B.1.617.3), and this neutralizing activity was similar to that toward the WT strain (IC_50_ = 2.65–9.82 ng/mL; Figure 1B). The results of the authentic virus assay showed that mAb 20D8 had adequate neutralizing activity against the WT, B.1.351, and P.1 variants of SARS-CoV-2 (IC_50_ = 9.6–22.4 ng/mL), and had relatively decreased neutralizing activity against the B.1.617.2 (Delta) variant (IC_50_ = 127 ng/mL; Figure 1C).

### 3.2. Protective Effect of mAb 20D8 in a Hamster Model

**Figure 2:**
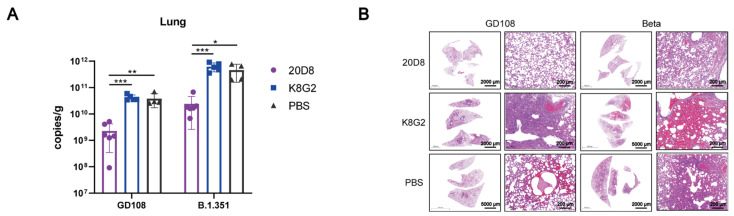
Protective effect of mAb 20D8 treatment in hamsters challenged with WT or a Beta strain of SARS-CoV-2. mAb 20D8 and negative control K8G2 (mAbEV71) were administered 1 day post-challenge and hamster lungs were collected and analyzed 5 days post-challenge. (**A**) Viral copy numbers in hamster lungs. One-way ANOVA was used to determine the statistical significance. *, *p* < 0.5; **, *p* < 0.05; ***, *p* < 0.005. (**B**) Pathological sections of the lungs of challenged hamsters following treatment with mAb 20D8, K8G2 (mAb EV71, negative control), or PBS (blank control).

Hamsters infected with SARS-CoV-2 WT or the Beta strain showed significantly reduced viral loads in lung tissue after treatment with mAb 20D8 compared to those of the negative EV71 mAb control group (K8G2) and the PBS control group (Figure 2A). Pathological analysis of animals challenged with the WT strain showed that hamsters in the EV71 mAb negative control group (K8G2) exhibited mild hemorrhage in the aorticopulmonary septum, infiltration of inflammatory cells, and local consolidation in lung tissues (Figure 2B). Hamsters in the PBS control group showed moderate hemorrhage in the aorticopulmonary septum and alveolar space, infiltration of inflammatory cells, thrombosis in the vascular lumen, thickening of the local pulmonary septum, and shedding of histiocytes in the bronchial lumen. However, no obvious histopathological changes were observed in the lungs of hamsters challenged with the WT strain and treated with mAb 20D8. Moreover, no obvious histopathological changes were observed in the lungs of hamsters challenged with the Beta variant and treated with 20D8, whereas severe hemorrhage in the pulmonary lobe and bronchial lumen was observed in the K8G2 control group. Dark staining lesions in the pulmonary lobe areas were observed in the PBS control group. The results showed that mAb 20D8 provided favorable protective effects against challenge with WT and Beta strains of SARS-CoV-2.

### 3.3. mAb 20D8 Recognizes a Linear Epitope (Amino Acids 369–379) of the RBD

**Figure 3:**
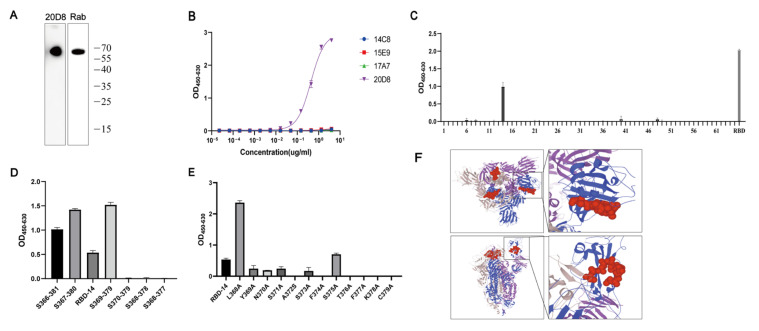
Specific binding of mAb 20D8 to the linearized RBD epitope (amino acids (aa) 369–379) of the spike (S) protein. (**A**) Western blot detection of the binding capacity of 20D8 to the linearized RBD (Rab: positive control). (**B**) Indirect ELISA detection of 20D8 recognition of the linearized RBD. (**C**) Testing of 20D8 binding to 65 polypeptides (12 aa long with a 9 aa overlap) that covered the RBD region (aa 329–537) of the S protein. (**D**) Based on RBD-14, six polypeptides were synthesized to further precisely locate the domain recognized by 20D8. (**E**) Alanine scanning of the effect of epitope amino acid mutations on 20D8 binding. (**F**) Spatial structure of the 20D8-recognized linear epitopes on S protein trimers.

Western blotting results showed that 20D8 exhibited the highest binding activity with the linearized RBD among the four mAbs tested (Figure 3A). Moreover, indirect ELISA, after linearization of the RBD, also confirmed that 20D8 could recognize linearized RBD (Figure 3B). This suggested that 20D8 was a neutralizing antibody against the linear epitope and that the recognized epitope was located in the RBD. To identify the specific 20D8 binding epitope, 65 polypeptides consisting of 12 amino acids (aa; with an overlap of 9 aa) were synthesized to cover the RBD region (aa 329–537) of the S protein. mAb 20D8 recognized polypeptide no. 14 (aa 368–379; Figure 3C). Six additional polypeptides were synthesized to precisely locate the domain recognized by 20D8. Enhanced mAb-polypeptide binding activity was observed when aa 368 was removed (aa 369–379), whereas 20D8 binding failed after the removal of aa 379 (aa 368–378) (Figure 3D). Mutation of L368 and S375 residues to alanine enhanced the binding capacity of 20D8, whereas mutations of A372S, F374A, T376A, F377A, K378A, and C379A led to a loss of binding capacity (Figure 3E). Therefore, the linear epitope recognized by 20D8 was determined to be YNSASF*TFKC. The location of this epitope in the spatial structure of the S protein (Protein Data Bank in Europe, 6 *×* 2b) is indicated in red in Figure 3F. The epitope consists of a random coil and half of a β-pleated sheet. When the RBD is in its “up” conformation, the epitope is exposed on the surface of the S protein trimer and it can be recognized by mAb 20D8, thus blocking the interaction of the virus with the Angiotensin-converting enzyme 2 (ACE2) receptor and preventing the virus from entering the cells.

### 3.4. ELISA

An ELISA method was used to determine the antigenic content of SARS-CoV-2 protein subunit vaccines, developed based on the above-mentioned concepts of USP <1220>, ICH Q14, and Q2 (R2). A porcine anti-S polyclonal antibody was used as the coating antibody in each plate well. The monoclonal antibody 20D8-HRP with RBD binding activity, broad-spectrum anti-mutant neutralizing activity, better in vivo protection, and clear epitope recognition obtained in this study was used as the detecting antibody for ELISA.

#### 3.4.1. Specificity

Recombinant S proteins of SARS-CoV and MERS-CoV (1 μg/mL, 1 μg/mL), MEM, DMEM, FBS, and PBS tested negative (OD value < cut-off value), whereas the ELISA result was positive for the NS (SARS-CoV-2 S protein, 0.4 U/mL). These results support the high specificity of the assay.

#### 3.4.2. Risk Assessment

**Figure 4:**
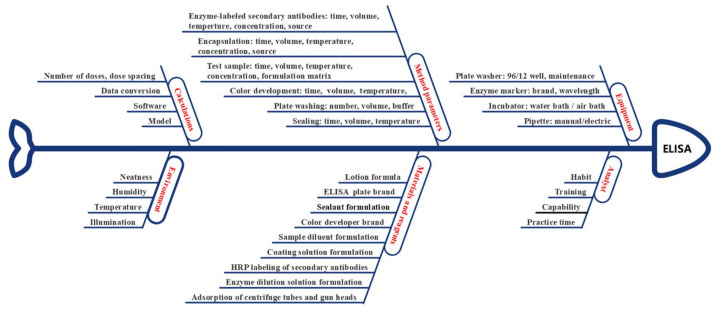
Ishikawa diagram summarizing various potential influencing factors on ELISA. Input factors are categorized into six main groups: equipment, analysts, materials and reagents, method parameters, environment, and calculations.

The various factors influencing the ELISA are visualized in an Ishikawa diagram in Figure 4. A failure mode effect analysis (FMEA) table was created to rank the individual influencing factors (Table 1). Depending on the degree of influence of each factor on the accuracy, precision, and specificity of ATP, five levels were assigned (1, 2, 3, 4, and 5). For example, the operational ability of analysts had a high impact on the experiment, a very high potential impact on robustness (score = 4) (priority = 10), a high estimated impact on accuracy (score = 4) (priority = 8), and a relatively low impact on specificity (score = 1) (priority = 8), resulting in a total risk score of 80. Based on the risk scoring of the influencing factors, the factors that had a more significant impact on the experiment (analytical procedure parameters) were screened from among those with total scores higher than 50. A robust DoE was effectively used [31].

#### 3.4.3. Screening of Fixed Factors

Based on the results in Section 2.6, the fixed factors’ concentration corresponding to the highest signal-to-noise ratio (S/N value) was selected (S/N value = 51). The optimal concentration of the coating antibody was determined to be 1 μg/mL, and the optimal working concentration of the enzyme-labeled mAb (HRP-20D8) was 0.25 μg/mL (Figure S2).

#### 3.4.4. Optimization

Monte Carlo simulations were performed based on the experimental data presented in Section 2.6 (Figure S1). The Monte Carlo method is the most frequently applied method for evaluating model uncertainty [10]. ANOVA was performed to evaluate the significance of the terms of the model. The results showed that the selected model of the factors was statistically significant (*p* < 0.05; see Table S2).

Incubation and color development were performed at 37 °C. Antigen and antibody incubation times were screened over a period of 45–120 min, and color development time was screened over a period of 10–30 min. Monte Carlo simulations were performed for 10,000 experiments, and the probability of experimental failure was guaranteed to be 0. The above ideal control space (incubation temperature, color development temperature, antigen incubation time, antibody incubation time, and color development time) was determined to be the MODR. By selecting an optimal working point within the MODR, this experiment was guaranteed to be carried out properly with a further reduction in the experiment time. Therefore, the incubation color development temperature was set to 37 °C, the incubation time for both the antigen and antibody was 1 h, and the color development time was 15 min (Figure S3).

Based on the analysis of the above results, an ELISA was established and optimized using mAb 20D8. The optimal concentration of the coating antibody was 1 μg/mL and that of the enzyme-labeled mAb (HRP-20D8) was 0.25 μg/mL. The blocking solution was PBS containing 5% FBS. The plates were coated at 2–8 °C overnight and then blocked at 37 °C for 2 h. The incubation time for both the antigen and antibody was 1 h, and color development was carried out at 37 °C for 15 min.

#### 3.4.5. Model Selection

After determining the optimal experimental conditions for the ELISA, the resulting data (based on Section 2.6) were fitted to linear, double log-linear (x-logarithmic and y-logarithmic), and four- or five-parameter logistic models. The precision, accuracy, and misjudgment probability of the method (MMJP) are shown in Tables S3–S5, respectively. The precision and accuracy of the double log-linear fit and five-parameter logistic fit were >92%, and the MMJP was <1% (corresponding to the concentration range). The double log-linear fit and a five-parameter logistic model were considered adequate. However, the five-parameter logistic model did not complete the “S” curve in the 0.016–1 U/mL concentration range. Therefore, a double log-linear model was selected for further analysis.

### 3.5. Validation of Method

#### 3.5.1. Range

**Figure 5:**
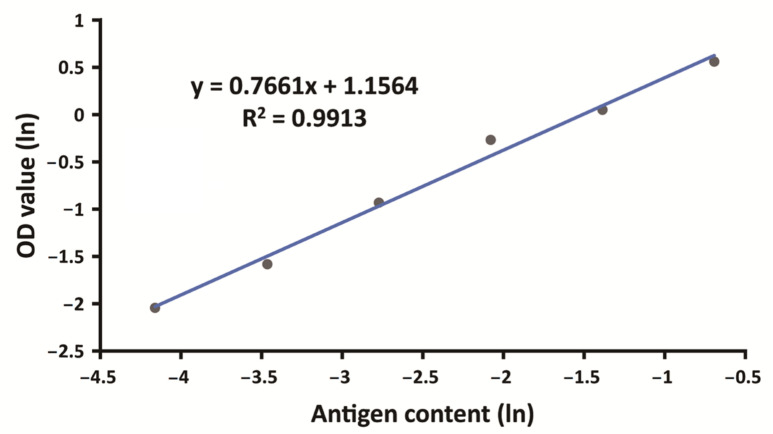
Detectable range of the ELISA for the NS antigen. Linearity was tested using 16 replicates; representative results of one test are shown here.

A double-log-linear model was used to validate the detection range of the NS, which was 0.016–0.5 U/mL (Figure 5).

#### 3.5.2. Accuracy and Precision

Based on USP <1033>, validation test samples may be constructed using a dilution series of the NS to assess dilutional linearity (linearity of the relationship between known and measured relative potency) [13]. Here, accuracy and precision were greater than 90% for all NS concentrations (Table 2). The pre-set values for ATP were met, and the accuracy and precision of the method met these requirements.

#### 3.5.3. Sample Size Evaluation

According to the experimental results presented in Section 3.5.2, the accuracy and precision of the method were ≥90%. Using the individual means for the sample size in the JMP 13^®^ software analysis, the required sample size for the method validation experiment was calculated to be >13. Our sample size of 16 met the requirements, and the probability of obtaining robust results was 96.19%.

#### 3.5.4. Method Capability Evaluation Indicators of ELISA

The overall method capability, accuracy, and precision of the ELISA were evaluated. The results of BMV software calculations are presented in Table 3. The relative prediction and tolerance intervals corresponding to NS concentrations ranging from 0.50 to 0.016 U/mL were in the range of 71–141%. Within this range, the method capability indices (MCI) all reached grade IV or higher, and the total method error did not exceed 11.83%. Based on relevant studies, the specification range of the method was determined to be 70–143%, and the probability of misjudgment of the method was <0.39% [32]. This method satisfies the expected requirements.

The ATP of this method was initially set based on experimental experience (Table 4). The final ATP settings are listed in Table 4. The intended purpose of this method was to establish the antigen content of the SARS-CoV-2 protein subunit vaccine and thus to perform release testing of such products. Accuracy and precision were ≥90%, which met the requirements of the ATP setting. When the specification range was 70–143%, the method capability index was >0.96, and the misjudgment probability of the method was <0.39%, which also met the requirements of the ATP setting.

### 3.6. Method Suitability Evaluation of ELISA

**Figure 6:**
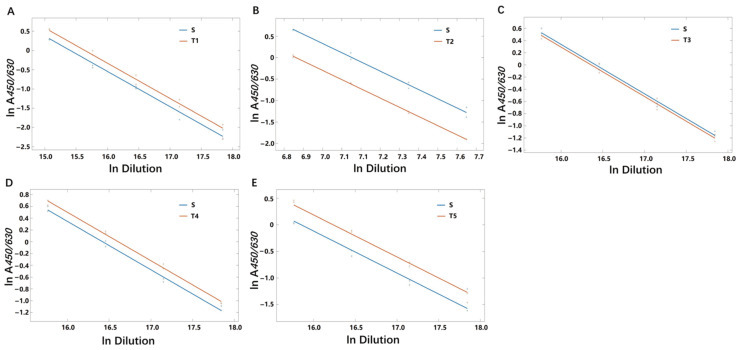
Parallelism and linearity in method suitability evaluation. (**A**–**E**) ELISA detection of the S or RBD protein in five different manufacturers’ bulks of SARS-CoV-2 recombinant protein vaccine. Each group was able to ensure regression term variation *p* < 0.05, lack of fit term variation *p* > 0.05, and deviation from parallel *p* < 0.05. S: NS; T1, T2, T3, T4, T5: the protein subunit vaccine stock solution of the SARS-CoV-2 variants developed by five manufacturers.

To ensure that the ELISA method established in this study could be used reliably in SARS-CoV-2 protein subunit vaccines from different VOCs, NIFDC carried out the tests with the bulks of protein subunit SARS-CoV-2 vaccines against the NS using the ELISA method. Five bulks (two S proteins and three RBD proteins) were prepared based on the RBD or S protein sequences of the Alpha, Beta, Gamma, and Delta variants of SARS-CoV-2. Four bulks were expressed in CHO cells, and one in Sf9 cells (Table S6). The results showed adequate parallelism and linearity for each bulk compared with NS (Figure 6). This indicated that the ELISA method is suitable for the detection of SARS-CoV-2 protein subunit vaccine S and RBD proteins of Alpha, Beta, Gamma, and Delta variants.

## 4. Discussion

The WHO and national vaccine regulatory agencies face great challenges in conducting regulatory science research and promoting vaccine development. Robust and reliable testing methods help guarantee product quality. Accurate and reliable detection of antigen content in SARS-CoV-2 protein subunit vaccines is not only relevant to the assessment of the consistency of product quality, but also for vaccine process optimization and clinical dose selection [3,5,7]. SARS-CoV-2 protein subunit vaccines constitute the highest proportion (32%) of vaccines in clinical trials globally [6]. Several Chinese manufacturers are stepping up their research and development in this area. One manufacturer has received conditional marketing approval for its SARS-CoV-2 protein subunit vaccine, and eight are in the process of conducting clinical trials [3]. In this study, an accurate, reliable, and universal method for the quantitative detection of vaccine antigens was developed based on concepts related to AQbD and modern methodological system theory [12,13,14,15,16,17]. The role of regulatory science can be fully utilized to promote the development of a new generation of SARS-CoV-2 vaccines.

Optimization of antibody selection formed the basis for the development of the ELISA in this study. The primary antibody selected for this study, mAb 20D8, exhibited high and broad-spectrum RBD binding activity and broad-spectrum neutralizing ability of VOCs (EC50: 2.7–9.8 ng/mL for pseudoviruses; EC50: 9.6–127 ng/mL for authentic viruses). In addition, mAb 20D8 recognized the conserved linear epitope 369–379 aa of the SARS-CoV-2 RBD region, specifically sequence YNSASF*TFKC, which is part of the CR3022 site. Within this sequence, three residues (Y369, F377, K378) were determined to be included in the epitope identified by 20D8. Further investigation showed that A372, F374, T376, F377, K378, and C379 are the sites required for mAb 20D8 binding, and a mutation in any one of these residues leads to a loss in 20D8 binding capacity for RBD. When the RBD is in its “up” conformation, epitope 369–379 is more exposed, facilitating binding to mAb 20D8, which is consistent with the role of the CR3022 epitope [33]. Sequence comparison revealed that the epitope recognized by 20D8 in SARS-CoV-2 is different from the equivalent region in SARS-CoV and MERS, and 20D8 could be used as a detection mAb in a SARS-CoV-2 protein subunit vaccine antigen content assay. The SARS-CoV-2 S protein could be detected by 20D8, and there was no cross-reactivity with the S proteins of SARS and MERS, which are both coronaviruses. The 20D8 recognition epitope was highly conserved in the Alpha, Beta, Gamma, and Delta strains of VOCs, and the results confirmed the suitability of the method for detecting the antigens in five manufacturer-produced VOCs (designed in Alpha, Beta, Gamma, and Delta strains). The Omicron BA.1 strain has mutations in three amino acid sites (S371L, S373P, S375F) of the S369–379 epitope, resulting in a loss of mAb 20D8 binding and neutralizing ability. However, for the quantitative examination of currently marketed and emergency protein subunit vaccines, and in-process protein subunit vaccines containing antigens of pre-Omicron mutant strains, this method can meet the current needs of companies for accurate quantification of the antigen content of SARS-CoV-2 protein subunit vaccines. The method could be useful to overcome the sequence difference between Omicron and post-Omicron variants. This will be followed up with further use of an antibody that targets a new linear conserved S-protein/RBD epitope. This method also has many applications in future vaccine quality control.

The AQbD concept is clearly defined in USP <1220> for the analytical procedure life cycle, ICH (Q14), (Q2(R2)), and other guidelines issued in 2022. In these guidelines, new terms, such as ATP, risk assessment, CMA, analytical procedure parameters, MODR, and enhanced models, are explained in a scientific and standardized manner. ELISA is one of the most commonly used analytical biochemistry assays to quantify levels of a specific target within a sample [34,35,36,37]. During the development of the ELISA method described here, the modern methodological system theory of detection was applied. Combining the above concepts with practical examples ensures that the approach meets the intended purpose throughout its life cycle. The early assessment phase focused on identifying high-risk factors. One of the most widely used risk assessment tools is the Ishikawa diagram. It enables the assignment of risks associated with factors to categories related to equipment, material, method, analyst, laboratory environment, etc. FMEA is another tool used to evaluate and prioritize factors to assess the severity of each risk [10]. Based on the Ishikawa diagram, FMEA, and prior knowledge, a set of potential influencing factors can be identified for DoE studies [38]. Using DoE for method development, the “optimal” setting can be achieved by evaluating influencing factors and their interactions. Therefore, a better understanding of the method can be achieved, and the robustness of the analysis methods can be studied [21,39]. For example, for the first time, indicators of method capability were given, such as the method of prediction interval, tolerance interval, method capability index, and misjudgment probability of the method [40,41,42]. In addition, these metrics are more intuitive and easier to understand, making them appealing to researchers and regulators. Previous studies have reported the development of biological assays based on the QbD concept [28,43]. Many researchers are already aware of the AQbD concept but have not yet applied it to experiments. Hopefully, AQbD will be more commonly integrated in future studies. The method successfully detected SARS-CoV-2 protein subunit vaccine antigens (RBD or S protein designed in Alpha, Beta, Gamma, or Delta sequences) obtained from five different manufacturers. Notably, this method cannot detect differences in sequence, expression systems (e.g., post-translational modifications), or functional neutralization activity of the expressed proteins.

In this study, the AQbD concept was successfully applied, providing a scientifically robust method for ELISA detection of the antigenic content of SARS-CoV-2 protein subunit vaccines. This is in line with the development concept of regulatory science and is of great significance in accelerating vaccine development and application. At the same time, as a case study of the application of a new concept, this study fully demonstrates the advanced and scientific nature of the AQbD concept and innovative nature of the other evaluation indicators. These new concepts can also be extended to other biological products and play a leading role in research on their quality control methods. This helps improve the concept related to the development of biological assays.

## Figures and Tables

**Table 1:** Failure mode effect analysis (FMEA).

Rating of Importance (Priority)			8	8	10	
ID	Process Step	Process Input	Accuracy	Specificity	Precision	Total
1	Environment	Temperature	1	1	1	26
2	Environment	Illumination	1	1	1	26
3	Environment	Humidity	1	1	1	26
4	Environment	Neatness	1	1	1	26
5	Enzyme markers	Brand	1	1	1	26
6	Plate washer	96-well/12-well	1	1	1	26
7	Calculation	Software	1	1	1	26
8	Enzyme marker	Wavelength	2	1	1	34
9	Blocking	Volume	1	2	1	34
10	Test sample	Volume	2	1	2	44
11	Enzyme-labeled mAbs	Volume	2	1	2	44
12	Substrate	Volume	2	1	2	44
13	Enzyme labeling plate	Brand	2	1	2	44
14	Substrate	Brand	2	1	2	44
15	Analyst	Habit	2	1	2	44
16	Analyst	Training	2	1	2	44
17	Analyst	Practice time	2	1	2	44
18	Consumable	Absorbance	2	1	2	44
19	Blocking	Volume	2	1	2	44
20	Coating	Volume	1	1	3	46
21	Blocking	Time	2	2	2	52
22	Blocking	Temperature	2	2	2	52
23	Coating	Temperature	2	1	3	54
24	Calculation	Model	3	1	3	62
25	Pipette	Electric/manual	3	1	3	62
26	Washboard	Buffers	2	3	3	70
27	Lotion	Recipe	2	3	3	70
28	Sample dilution	Recipe	2	3	3	70
29	Enzyme secondary antibody dilution	Recipe	2	3	3	70
30	Blocking solution	Recipe	2	3	3	70
31	Coating solution	Recipe	2	3	3	70
32	Test sample	Concentration	3	1	4	72
33	Test sample	Time	3	1	4	72
34	Test sample	Temperature	3	1	4	72
35	Enzyme-labeled mAbs	Concentration	3	1	4	72
36	Enzyme-labeled mAbs	Time	3	1	4	72
37	Enzyme-labeled mAbs	Temperature	3	1	4	72
38	Coating	Concentration	4	1	4	80
39	Coating	Time	4	1	4	80
40	Washboard	Number of times	2	3	4	80
41	Calculation	Concentration point setting	4	1	4	80
42	Incubator	Incubation mode	4	1	4	80
43	Analyst	Capability	4	1	4	80
44	Substrate	Time	4	3	5	106
45	Substrate	Temperature	4	3	5	106
46	Plate washer	Maintenance	5	5	5	130

**Table 2:** Analysis of accuracy and precision results (*n* = 16).

Antigen Content (U/mL)	0.50	0.25	0.13	0.063	0.031	0.016
Bias (%)	−8.48	4.51	6.55	4.51	−1.50	−4.86
Confidence interval for bias (%)	−11.57−5.28	2.30−6.77	4.40−8.75	2.40−6.66	−3.03−0.061	−7.49−2.15
Intermediate precision (%)	8.25	5.50	5.12	5.39	4.45	7.29
Upper bound on intermediate precision (%)	12.01	7.66	7.19	7.44	6.01	10.18

**Table 3:** Evaluation of method capability at different concentration levels.

Antigen Content (U/mL)	Method Variability (%)	90% Tolerance Interval (%)	90% Prediction Interval (%)	MCI Under	Misjudgment Probability of Method	Method Level
0.50	11.83	70.07−119.54	73.83−113.46	0.96	3.92 × 10^−3^	VI
0.25	7.11	89.43−122.14	92.20−118.47	1.65	7.62 × 10^−7^	II
0.13	8.31	88.88−127.73	92.09−123.28	1.42	2.14 × 10^−5^	II
0.063	7.03	89.59−121.90	92.33−118.29	1.67	5.64 × 10^−7^	I
0.031	4.70	88.81−109.25	90.63−107.06	2.48	1.02 × 10^−13^	I
0.016	8.76	78.39−115.47	81.42−111.18	1.33	6.94 × 10^−5^	III

MCI: Method capability indexes.

**Table 4:** Analytical target profile (ATP) and comparison with final development results.

Antigen Content (U/mL)	(A) Development Objectives	(B) Development Results
Intended purpose	Quantifying the antigen content of SARS-CoV-2 recombinant protein vaccines.	
Link to critical quality attribute (CQA)	CQA: The antigen content of SARS-CoV-2 recombinant protein vaccines. Experimental principle: The specific antibody was bound to a solid-phase carrier to form a solid-phase antibody. The immune complex was then formed by binding to the corresponding antigen in the sample to be examined. After washing, enzyme-labeled antibodies were added and bound to antigens in the immune complexes to form enzyme-labeled monoclonal antibody–antigen solid-phase complexes. The antigen content was determined by adding substrate for color development. (The shade of color in the microplate was positively correlated with the concentration of the substance to be measured.)	
Specificity	No cross-reactivity with SARS-CoV and MERS-CoV recombinant S proteins.	No cross-reactivity with SARS-CoV and MERS-CoV recombinant S proteins.
Accuracy	≥85%	≥90%
Precision	≥85%	≥90%
Total analytical error	≤20%	≤11.83%
Specification range to be controlled by this method	63−158%	70−143%

Note: Form (A) was developed based on prior experience before analysis development. After the method development was completed, form (B) was developed based on the validation results.

## Data Availability

Not applicable.

## Supplementary Materials

The following supporting information can be downloaded at: https://www.mdpi.com/article/10.3390/v15010062/s1, Figure S1: Experimental design for method validation; Figure S2: Heat map of fixed factors; Figure S3: Screening for optimal ELISA experimental conditions; Table S1: Experimental schedule; Table S2: Evaluation of experimental design model parameters; Table S3: Comparison of the precision between models; Table S4: Comparison of the accuracy between models; Table S5: Comparison of MMJP between models; Table S6: Information of test samples.
