# Supplementary material for: Development of an ELISA Assay for the Determination of SARS-CoV-2 Protein Subunit Vaccine Antigen Content
Source: Viruses. 2022 Dec 24;15(1):62. doi: 10.3390/v15010062 (PMC9860593; doi:10.3390/v15010062)
Supplement: Supplementary file 1 [file viruses-15-00062-s001.zip › Supplementary_materials.pdf]

Supplementary Materials

Table S1. Experimental schedule

Test	Incubation temperature (°C)	Color development temperature (°C)	Antigen incubation time (min)	Antibody incubation time (min)	Color development time (min)	S/N	N
1	25±2	37	30	120	20	19.3	0.052
2	25±2	37	75	30	30	28.7	0.052
3	25±2	37	120	120	30	50.1	0.048
4	25±2	37	30	75	10	10.1	0.043
5	25±2	37	120	30	20	19.7	0.052
6	37	37	30	120	10	24.1	0.050
7	37	37	30	120	30	28.2	0.062
8	37	37	120	30	10	26.7	0.048
9	37	37	120	30	30	49.0	0.049
10	37	37	75	75	20	43.8	0.053
11	25±2	25±2	75	120	10	31.5	0.046
12	25±2	25±2	120	75	30	40.5	0.042
13	25±2	25±2	30	30	20	5.13	0.133
14	25±2	25±2	120	75	10	28.5	0.046
15	25±2	25±2	75	120	30	44.0	0.058
16	37	25±2	75	75	20	44.0	0.051
17	37	25±2	120	120	30	51.0	0.057
18	37	25±2	120	120	10	45.3	0.054
19	37	25±2	30	30	30	29.3	0.047
20	37	25±2	30	30	10	13.1	0.053
21	25±2	37	75	30	10	16.5	0.048
22	25±2	37	30	30	30	18.6	0.049
23	25±2	37	120	120	20	50.3	0.049
24	25±2	37	30	75	30	14.1	0.044

S/N: signal-to-noise ratio; N: noise.

Table S2. Evaluation of experimental design model parameters

Observed criteria	Regression ANOVA statistics	
S/N	R^2 = 0.9943<br>Adjusted R^2 = 0.9737	p=0.0002
N	R^2 = 0.9848<br>Adjusted R^2 = 0.9300	p=0.0023

A p-value < 0.05 demonstrated that at least one of the factors had a significant effect on the model, and the model was valid. S/N: signal-to-noise ratio; N: noise.

Table S3. Comparison of the precision between models

Fitting model	Antigen concentration (U/mL)						
	0.0156	0.0313	0.0625	0.125	0.25	0.5	1
Linear	10.82	2.97	1.72	3.81	0.84	/	/
Double log-linear	1.28	3.03	3.92	2.53	2.19	1.47	/
Five-parameter logistic	4.22	2.89	4.46	2.12	1.85	1.18	3.61
Four-parameter logistic	25.82	3.87	3.60	1.84	2.22	1.70	2.18

/ = not tested.

Table S4. Comparison of the accuracy between models

Fitting model	Antigen concentration (U/mL)						
	0.0156	0.0313	0.0625	0.125	0.25	0.5	1
Linear	−27.69	−6.20	5.38	5.16	−1.42	/	/
Double log-linear	−3.71	−1.87	4.17	5.61	3.36	−6.92	/
Five-parameter logistic	2.52	−2.45	−0.03	0.43	−0.12	0.09	0.15
Four-parameter logistic	−32.44	1.74	6.84	0.95	−3.13	2.40	−1.04

/ = not tested.

Table S5. Comparison of MMJP between models

Fitting model	Antigen concentration (U/mL)						
	0.0156	0.0313	0.0625	0.125	0.25	0.5	1
Linear	0.48	0.0017	0.00024	0.00028	1.56×10^{-41}	/	/
Double log-linear	2.268×10^{-8}	2.643×10^{-10}	6.921×10^{-5}	2.009×10^{-4}	1.590×10^{-8}	2.308×10^{-3}	/
Five-parameter logistic	3.810×10^{-6}	3.310×10^{-9}	3.065×10^{-7}	1.692×10^{-25}	5.720×10^{-34}	7.628×10^{-81}	3.113×10^{-10}
Four-parameter logistic	6.234×10^{-1}	8.894×10^{-8}	2.934×10^{-3}	1.400×10^{-27}	7.618×10^{-9}	1.590×10^{-14}	1.462×10^{-20}

/ = not tested. MMJP: Misjudgment probability of method.

Table S6. Information of test samples

Test sample	Host cell	Protein	Measured concentration	Labeled concentration
A	CHO	RBD	0.5 YU/ml	1055,000 YU/ml
B	Sf9	RBD	1 CU/ml	910,000 CU/ml
C	CHO	S	32 ng/ml	403,200 ng/ml
D	CHO	S	32 ng/ml	800,000 ng/ml
E	CHO	RBD	0.9 ng/ml	346,000 ng/ml

A-D：represent four different test samples.

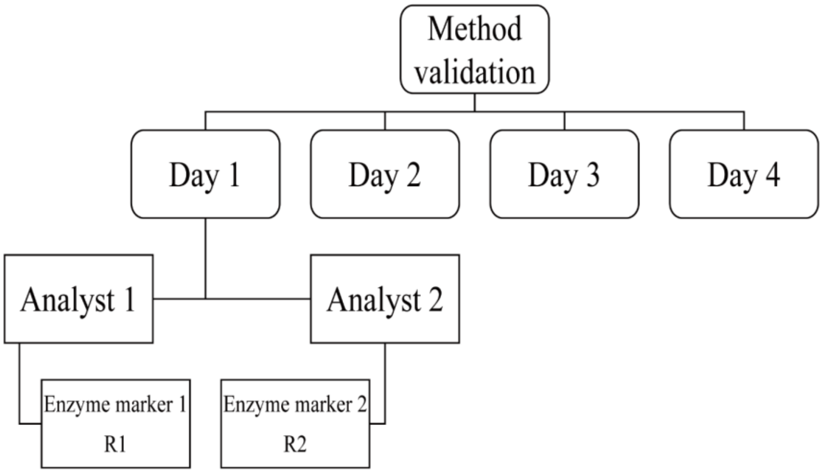

Figure S1. Experimental design for method validation. The method is validated over 4 days (Day 1–4) by two analysts (Analyst 1 and 2). Each analyst uses two enzyme markers (Enzyme marker 1 and 2). R1 and R2 represent two independent experiments.

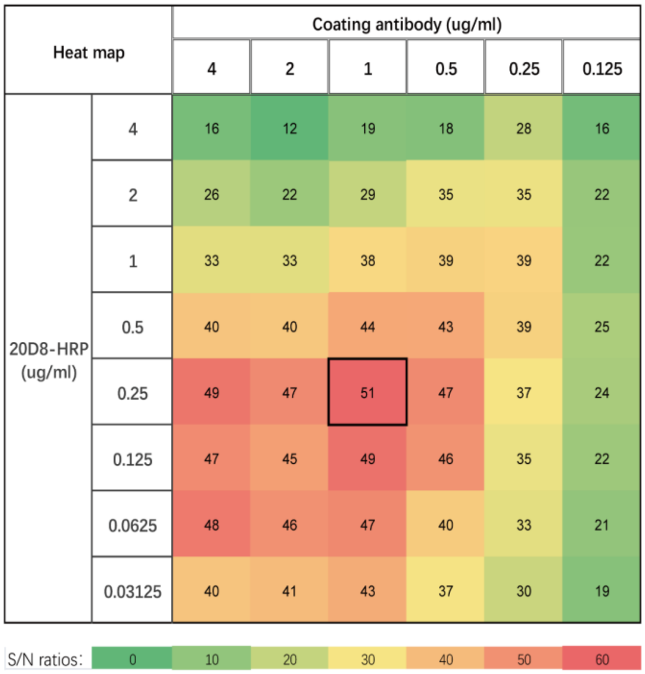

Heat map		Coating antibody (ug/ml)					
		4	2	1	0.5	0.25	0.125
20D8-HRP (ug/ml)	4	16	12	19	18	28	16
	2	26	22	29	35	35	22
	1	33	33	38	39	39	22
	0.5	40	40	44	43	39	25
	0.25	49	47	51	47	37	24
	0.125	47	45	49	46	35	22
	0.0625	48	46	47	40	33	21
	0.03125	40	41	43	37	30	19

Figure S2. Heat map of fixed factors. The classification was based on the signal-to-noise ratio (S/N) value, which was divided into seven grades (i.e., seven colors) from 0–60. The condition resulting in the highest S/N value is marked. S/N: signal-to-noise ratio; N: noise.

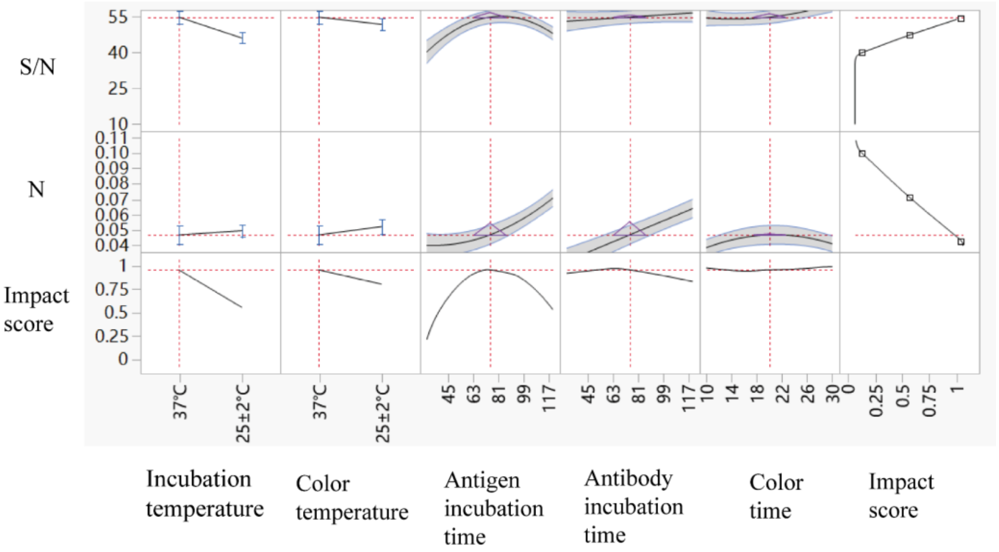

Figure S3. Screening for optimal Elisa experimental conditions. The dynamics of each influencing factor (i.e., incubation and color development temperature, and antigen, antibody, and color development time) were visualized using Monte Carlo simulations. The purple triangle indicates the sensitivity; the higher the triangle, the more significant the impact of a factor on the experiment. “Impact score” takes the response importance weight into account; the closer to 1, the better the impact score. S/N: signal-to-noise ratio.
